# Determination of the botanical origin of honeybee honeys based on the analysis of their selected physicochemical parameters coupled with chemometric assays

**DOI:** 10.1007/s10068-019-00598-5

**Published:** 2019-04-06

**Authors:** Ewa Majewska, Beata Drużyńska, Rafał Wołosiak

**Affiliations:** Division of Food Quality Evaluation, Department of Biotechnology, Microbiology and Food Evaluation, Faculty of Food Sciences, Warsaw University of Life Sciences – SGGW, 159 Nowoursynowska Str., 02-787 Warsaw, Poland

**Keywords:** Honey, Ash, Electrical conductivity, Chemometric, Botanical origin

## Abstract

The aim of this paper was to study the select physicochemical parameters of 58 honey samples of 4 different botanical origins (buckwheat, linden, rape and acacia) using multivariate methods in order to classify honeys according to the botanical origin. Five standard physicochemical parameters were determined according to the international legislation: water content, electrical conductivity, total ash content, free acidity and pH. The results obtained were mostly in agreement with international regulations. Then, the results obtained were analysed by principal components analysis and cluster analysis. The chemometric analysis of results of determinations of the physicochemical parameters demonstrated such markers as electrical conductivity and ash content (i.e. parameters linked with minerals content) to be the most reliable markers in determining the botanical origin of linden and buckwheat honeys. Unfortunately, they appear insufficient for reliable identification of acacia and rapeseed honeys.

## Introduction

Honey is the natural sweet substance produced by *Apis mellifera* bees from the nectar of plants or from secretions of living parts or excretion of plant-sucking insects on the living parts of plants, which the bees collect, transform by combining with specific substances of their own, deposit, dehydrate, store and leave in honeycombs to ripen and mature (Council EU, 2001). Honey contains more than 200 substances, including amino acids, enzymes, protein, vitamins, minerals, ash, organic acids and phenol compounds (Bueno-Costa et al., 2016). Properties and compositions of bee honey depend strongly on the type of flowers the bees visited, season, on the climatic conditions in which the plants grow and treatment of beekeepers (El Sohaimy et al., 2015).

Botanical authenticity is one of the key problems in honey quality control because it directly affects its market price. Regulating bodies, food industry, retail sellers, and consumers are interested in knowing the origin and quality of honeys available on the market. Investigations on honey authenticity are conducted in many countries worldwide. One of the first methods used to determine the botanical and geographical origin of honeys was pollen analysis (El Sohaimy et al., 2015). However, this technique is time consuming and requires special personnel skill (Jandrić et al., 2015; Popek et al., 2017; Wang and Qing, 2011). For this reason, attempts are undertaken to introduce other analytical methods into the identification process of the origin of honeybee honeys. Hence, determination of the physicochemical parameters conducted in the routine assessment of honey is the most common method for detection of is origin (Juan-Borrás et al., 2014). Studies are underway on the use of various combinations of these parameters that would allow for determining the botanical origin of different types of honeys (Karabagias et al., 2014; Rodríguez Flores et al., 2015). To enable evaluation of honey authenticity, works are in progress to develop practical alternative methods and markers for determination of the botanical and geographical origin of this product.

Considering that many different compounds have been detected in honey, its evaluation should be perceived as a multi-variate analysis. Therefore the coupled use of physicochemical parameters and chemometric analyses is a method applied to determine the origin of both honey as well as other food products (Oroian et al., 2015; 2016). The use of chemometry in this case results from the fact that it is a discipline of science and technology of extracting useful information from multi-dimensional measurement data, using statistical and mathematical methods. It differs from the statistics not only in the character of the analyzed variables, but also in the mode of their analysis. Typical statistical methods have been developed to enable the analysis of single variables or of a small number of variables. By principle, the chemometric approach assumes the simultaneous analysis of many variables. Chemometric methods are increasingly often applied to identify natural groups of variables based on their similarity with analytical samples. There are many chemometric techniques of classification, the most common of which include: principal component analysis (PCA), linear discrimination analysis (LDA), cluster analysis (CA) (Juan-Borrás et al., 2014; Oroian et al., 2015; 2016), artificial neutral network (ANN), adaptive neutron-fuzzy inference (ANFIS) (Oroian 2015) and Classification & Regression Tree (C&RT) method for data exploration (Popek et al., 2017). The use of these methods allows reducing the complexity of large data sets and offers their better interpretation and understanding.

The objective of this study was an attempt of coupling determinations of parameters linked with the content of mineral compounds and chemometric analysis to identify the botanical origin of honeybee honeys.

## Materials and methods

### Chemicals

 The potassium chloride (KCl) and sodium hydroxide (NaOH) were obtained from Avantor Performance Materials Poland S.A. (Gliwice, Poland).

### Samples

A total of 58 honey samples were used for this study. Honey samples were directly obtained in 2015 from the private producers located in the whole territory of the Republic of Poland. The honey samples with different production origin and varieties have been collected. The variety of types of nectar honeys had been investigated: rape, buckwheat, willow and acacia. The botanical origin of honey samples were verified using a pollen analysis with the use of method established by the International Commission of Bee Botany. All samples were immediately transferred to the laboratory and kept stored unpasteurised at 4–5 °C in the dark.

### Determination of moisture

Water content (moisture) was determined based on the refractometric method using an Abbé-type refractometer (PZO, Warsaw, Poland) and the readings were further corrected for a standard temperature of 20 °C by adding the correction factor of 0.00023/°C. The honey samples that were crystalized at the moment of analysis were previously heated until 40 °C. Moisture content was determined in triplicate and the % moisture content values corresponding to the corrected refractive index values were calculated using Wedmore table.

### Determination of electric conductivity

Electrical conductivity (EC) of a honey solution at 20 g/100 g (dry matter basis) in CO_2_—free-deionised water was measured at 20 °C in a Crison Basic 30 conductimeter (Crison Instruments S.A., Barcelona, Spain). The instrument was calibrated using 0.01 M KCl. The results were expressed as S/cm. All measurements were performed in triplicate.

### Determination of ash content

Total ash content was determined according to the method of incineration of honey samples. About 5 g of honey was placed in combustion pots, which required preheating to darkness in electric cooker to prevent honey foaming. Then, the samples were incinerated at a temperature of 550 °C in a muffle furnace (Carbolite Gero Ltd., Hope Valley, United Kingdom) for 18 h. After cooling at room temperature, the obtained ash was weighed. All measurements were performed in triplicate.

### Determination of acid and pH parameters

Free acidity was determined by potentiometric titration. Honey samples were homogenized in a water bath before analysis. About 10 g of honey were dissolved in 75 mL of CO_2_—free distilled water. The solution was titrated with 0.05 mol/L NaOH to pH 8.30. Free acidity was determined in triplicate, The results were expressed in miliequivalent of acid per kilogram of honey. pH measurements were performed potentiometricaly a 20 °C using a pH-meter HANNA HI 931400 (HANNA Instruments, Woonsocket, Rhode Island, USA) in a 10% (w/v) solution of honey prepared with CO_2_—free distilled water. All measurements were performed in triplicate.

### Statistical analysis

Analyses were made in triplicate and the data are presented as mean ± standard deviation. Correlations were obtained by Pearson’s correlations between total ash and electrical conductivity. Multivariate analysis [principal component analysis (PCA) and cluster analysis (CA)] was performed by using STATISTICA version 13.1 (StatSoft Poland). Prior the chemometric processing the data matrix were standardised.

To classify honeys according to the physicochemical parameters, cluster analysis was applied. This method is frequently used to screen data for clustering of samples. The main goal of the hierarchical agglomerative cluster analysis was to spontaneously classify the data into groups of similarity (cluster), searching objects in the n-dimensional space located in the closest neighborhood and to separate a stable cluster from other clusters (Simeonov et al., 2007). Cluster analysis was displayed in order to find similarities between the honey samples and also between the variables. Then we were able to check if on that basis they create clusters and we could observe any similarities. In order to do that, Ward’s hierarchical cluster method for pattern recognition was used.

Using cluster analysis it was possible to display the object similarity in a reliable way and make the initial interpretation of the data set structure. But a more reliable method proved to be PCA. PCA was used to achieve a reduction of dimension, covering the maximum amount of variability present in the data and to observe a primary evaluation of the between-class similarity. This method provides a new variable as linear combinations of the original descriptor, which are called principal components (Simeonov et al., 2007). Principal component analysis was used as a descriptive tool to visualize the data in two dimensions, founding relationships between the variables and the type of honey. Classical principal component analysis on covariance matrix was used. It was used to search data trends and to provide a partial view of the data in a space with reduced number of dimensions while preserving most of their variability.

## Results and discussion

The results from the physicochemical analysis for the fifty-eight honey samples are presented in Table 1.

**Table 1:** Physico-chemical parameters of analysed honey samples (buckwheat = B, linden = L, rape = R and acacia = A)

Samples honey	Water content (%)	Electrical conductivity (S/cm)	Total ash content (%)	Free acidity (meq/kg)	pH
B1	16.7	367	0.12	70.8	3.81
B2	18.3	411	0.15	84.1	3.93
B3	19.0	746	0.37	71.2	4.07
B4	18.3	291	0.09	59.1	3.80
B5	18.6	380	0.13	67.4	3.82
B6	18.7	332	0.11	64.8	3.85
B7	17.5	400	0.15	91.3	3.78
B8	17.4	347	0.13	66.7	3.77
B9	19.3	428	0.17	87.4	3.78
B10	17.4	427	0.16	75.5	3.96
B11	19.5	110	0.04	28.7	3.80
B12	21.1	421	0.16	91.4	3.78
B13	19.5	499	0.25	61.2	4.02
B14	19.5	357	0.12	75.7	3.92
B15	18.1	429	0.16	65.0	3.89
B16	20.2	380	0.14	57.2	3.94
B17	19.8	290	0.08	42.8	4.20
Mean	18.8	389	0.15	68.3	3.89
SD	1.1	122	0.07	16.1	0.12
L1	19.5	226	0.05	34.7	3.87
L2	17.0	537	0.30	35.0	4.40
L3	19.8	450	0.22	50.8	4.09
L4	19.5	580	0.29	37.7	4.28
L5	17.9	552	0.26	45.0	4.10
L6	18.1	413	0.20	41.0	3.97
L7	21.1	284	0.09	27.0	4.33
L8	20.3	223	0.05	42.2	3.72
L9	19.3	89	0.01	24.1	3.94
L10	19.4	261	0.06	42.3	3.92
L11	18.1	529	0.26	35.5	4.34
L12	19.1	398	0.14	25.5	4.30
L13	19.7	488	0.20	28.7	4.38
L14	17.9	383	0.13	59.4	3.77
L15	19.9	558	0.26	38.5	4.22
Mean	19.1	398	0.17	37.8	4.11
SD	1.1	147	0.10	9.4	0.23
R1	18.7	194	0.08	25.9	4.09
R2	17.9	200	0.10	31.8	4.04
R3	19.3	172	0.06	26.4	4.19
R4	17.4	187	0.06	41.9	3.81
R5	19.0	182	0.06	28.9	4.22
R6	19.4	203	0.08	38.1	3.86
R7	19.9	263	0.10	37.8	3.94
R8	19.8	165	0.05	33.8	3.91
R9	18.6	135	0.03	31.2	3.80
R10	20.2	266	0.03	32.1	4.08
R11	19.5	159	0.02	33.0	3.93
R12	21.1	151	0.02	26.6	3.91
R13	17.8	187	0.05	33.7	3.98
R14	21.3	187	0.06	33.2	4.08
R15	20.2	197	0.08	37.2	3.88
R16	20.6	165	0.05	33.0	3.80
R17	18.3	242	0.14	41.7	3.94
R18	20.1	152	0.05	31.4	3.92
R19	19.3	312	0.17	38.8	3.96
R20	20.3	275	0.16	31.9	4.20
Mean	19.4	200	0.07	33.4	3.98
SD	1.1	47	0.04	4.6	0.14
A1	18.9	153	0.03	33.8	3.75
A2	19.0	179	0.07	29.1	3.95
A3	20.2	57	0.01	20.4	3.99
A4	18.5	199	0.05	40.7	3.81
A5	18.5	145	0.03	27.8	3.84
A6	20.1	198	0.10	28.1	3.78
Mean	19.2	155	0.05	30.0	3.85
SD	0.8	50	0.03	6.4	0.10

Water content of honey is deemed a very important parameter being indicative of improper storage conditions or of potential adulteration of honey in the production process (Fechner et al., 2016). On the other, moisture content may differ between honeys depending on the source of nectar or weather conditions in the region the honey is produced in. Therefore, values of this parameter may change from season to season and also from year to year. European (Council EU, 2001) and international standards (Codex Alimentarius, 2001) stipulate the maximum value of moisture content of honeys at 20% (w/w). Most of the honeys analyzed in our study had moisture content below 20%, but in 12 of them it was exceeded (Table 1). In the case of buckwheat honeys, these were samples B12 and B16 (21.1% and 20.2%, respectively); in the case of linden honeys—samples L7 and L8 (21.1% and 20.3%, respectively); in the case of rapeseed honeys—samples R10 (20.2%), R12 (21.1%), R14 (21.3%), R15 (20.2%), R16 (20.6%), R18 (20.1%) and R20 (20.3%); and in the case of acacia honeys—samples A3 and A6 (20.2% and 20.1%, respectively). These exceedings can result from weather conditions prevailing while acquiring honey and from its immaturity resulting from early acquiring. The higher water content may lead to undesirable fermentation of honey during its storage as a result of the activity of osmotolerant yeast synthesizing ethyl alcohol and carbon dioxide. Alcohol may be further oxidized to acetic acid and water, thereby contributing to the sour taste of honey (Fechner et al., 2016; Habib et al., 2014; Pita-Calvo and Vázquez, 2017).

Electrical conductivity of honey is strictly linked with contents of their mineral salts and organic acids (Grigoryan, 2016; Habib et al., 2014). It shows the greatest variability among the physicochemical parameters of honeys depending on their botanical origin and therefore enables discriminating between nectar and honeydew honeys (Pita-Calvo and Vázquez, 2017). The measurement of electrical conductivity is part of the routine control of honey and has recently replaced determination of ash content in the international (Codex Alimentarius, 2001) and European standards (Council EU, 2001), to finally become the most common method for honey origin identification (El Sohaimy et al., 2015). Values of electrical conductivity measured in the analyzed honeys and presented in Table 1 did not exceed the maximum value of 800 S/cm stipulated in the EU Directive (Council EU, 2001). In samples of buckwheat and linden honeys mean values accounted for 389 S/cm and 398 S/cm, respectively, whereas in samples of rapeseed and acacia honeys they were significantly lower and reached 200 S/cm and 155 S/cm, respectively. These results enable concluding that the electrical conductivity may be a reliable parameter for botanical discrimination of honeys. For example, acacia honey may be discriminated from the other honeys because it had the lowest mean value of this parameter.

Ash content of honey is indicative of the contents of its mineral compounds. It is claimed to be a criterion of honey quality and a parameter enabling discrimination between nectar and honeydew honey as the latter contains more minerals (Pita-Calvo and Vázquez, 2017). It is assumed that ash content of nectar honeys should be lower or equal to 0.6% (w/w) (El Sohaimy et al., 2015; Habib et al., 2014). Results obtained in our study (0.01% to 0.37%) (Table 1), fit within the above level, which may point to the purity of samples and probable lack of their adulteration with molasses.

Free acidity is a measure of honey condition deterioration. This parameter is linked with the natural presence of organic acids in honey, which remain in equilibrium with internal esters, lactones and some inorganic ions like: phosphates, sulfates and chlorides. In addition, a high value of total acidity may imply that at some point the honey began to ferment and that the produced alcohol was transformed into organic acids (Grigoryan, 2016). Pursuant to the Codex Alimentarius (2001) and EU Directive (Council EU, 2001), the permissible value of free acidity in honeys is at 50 meq/kg. Results of free acidity analysis were presented in Table 1. Its highest mean value was found in buckwheat honey (68.3 meq/kg). Out of 17 analyzed buckwheat honeys, only two did not exceed the maximum permissible value set in the international and European standards (B11—28.7 meq/kg—and B17—42.8 meq/kg). In the other honey varieties, the mean value of free acidity was lower than the permissible value and reached 37.8 meq/kg in linden honeys, 33.4 meq/kg in rapeseed honeys, and 30.0 meq/kg in acacia honeys. The above results allow concluding that the light honeys—including linden, rapeseed and acacia honeys—are characterized by a considerably lower content of organic acids compared to the dark honeys represented in our study by buckwheat honeys. Some authors (Fechner et al., 2016) suggest that free acidity might be used to discriminate nectar and honeydew honeys, however this was not confirmed by Oroian and Ropciuc (2017) who determined higher values of active acidity in nectar than in honeydew honeys.

Honey is an acidic food product which contains both organic acids and amino acids, and variability of its acidity which depends on its botanical origin may result from the presence of these two groups of chemical compounds (Oroian, 2012). The pH value of honey is correlated with its storage and with the growth of microorganisms capable of changing honey texture and stability. Although the EU Directive (Council EU, 2001) does not set any pH standards for honeys, its value should be low to prevent microbiological spoilage. The pH values of honeys usually range from 3.5 to 5.5 and are due to the presence of organic acid, gluconic acid in particular, and inorganic ions like phosphates and chlorides (Pita-Calvo and Vázquez, 2017). Results of pH measurements conducted in the analyzed honeys were presented in Table 1. The highest mean pH value (4.11) was determined for linden honeys. In turn, the mean pH value of buckwheat honeys accounted for 3.89, that of rapeseed honeys—for 3.98, and that of acacia honeys was the lowest and reached 3.85. The low pH value of honey determines its microbiological stability and may indicate high contents of minerals (El Sohaimy et al., 2015). This thesis was confirmed in our study because linden honeys had the highest pH value and the highest total ash content.

**Fig. 1:**
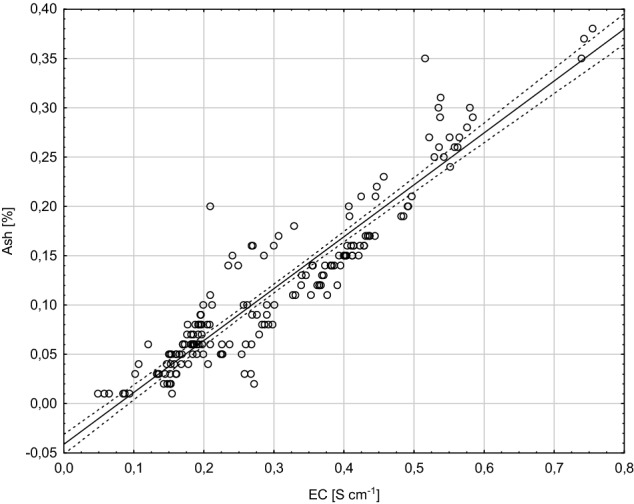
Correlation between electrical conductivity (EC) and ash content (confidence level 95%)

Electrical conductivity of honeys is strongly correlated with the concentration of their mineral salts, organic acids and proteins. It shows the greatest variability among the physicochemical parameters of honeys depending on their botanical origin (Fechner et al., 2016; Grigoryan, 2016). In our study, we were looking for a correlation between ash content and electrical conductivity of the analyzed nectar honeys, because conductivity corresponds well with content of minerals in honeys, i.e. the higher the ash content, the higher the conductivity is. This statement was confirmed by a correlation depicted in Fig. 1. In the case of the analyzed nectar honeys, the correlation coefficient reached 0.934, which is indicative of a very strong linear correlation between these two variables. The above results are consistent with literature data. A linear correlation (*r *= 0.99) was also found in our another research (Majewska and Kowalska, 2011) carried out with Polish nectar honeys. A linear correlation between ash content and electrical conductivity was also reported for Indian nectar honeys (*r *= 0.98) (Saxena et al., 2010). Such correlations between these two parameters has led to a situation in which electrical conductivity replaced ash content in the international (Codex Alimentarius, 2001) and European (Council EU, 2001) standards concerning evaluation of honeybee honey quality. At the same time, it has become the most frequently applied method for honey origin identification.

**Fig. 2:**
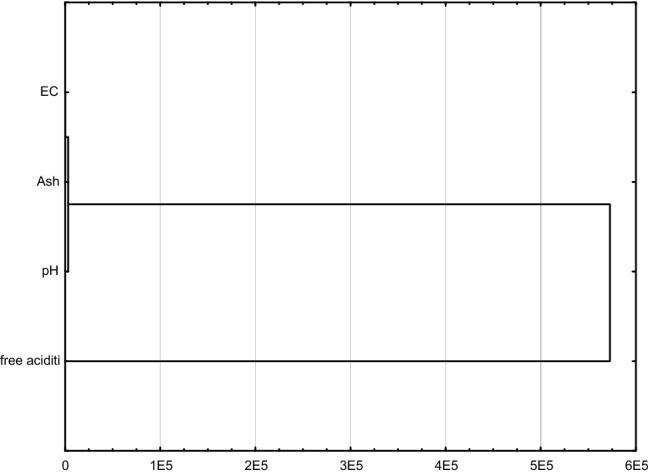
Dendrogram of variables (elements)

The cluster analysis (CA) conducted with the Ward method indicated botanical similarity of honey samples depending on their physicochemical parameters. The obtained dendrogram (Fig. 2), which depicts similarity of the analyzed physicochemical parameters, allowed distinguishing two clusters of parameters: the first one included electrical conductivity, ash content and pH of honeys, whereas the second one included only one parameter, namely content of free acids. Water content is a parameter which directly depends on many factors including weather conditions in the period of nectar harvest and degree of honey maturity. For this reason, this variable was not treated as a significant criterion in comparison of honeys originating from various plant sources.

Apart from the cluster analysis, we conducted also the principal component analysis (PCA). The appropriate number of principal components was selected based on the analysis of a scree plot made for the investigated honeys. It was found advisable to reduce four variables to two principal components that would together explain 93.6% of total variability of data. The first principal component (PC1) explained 59.45% while the second one (PC2) explained 34.15% of data variability. Values of coefficients of correlation (Table 2) between the variables and the principal components indicated that the first component (PC1) was strongly correlated with ash content and electrical conductivity of honey, and that the second component (PC2) was positively correlated with honey pH and negatively correlated with the content of free acids.

**Table 2:** Loadings of variables for the two PCs (principal components)—in bold are the significant loadings used for each principal component

Variable	PC1	PC2
EC	**0.986**	− 0.022
Ash	**0.959**	0.094
Free acids	0.554	− **0.783**
pH	0.424	**0.862**

**Fig. 3:**
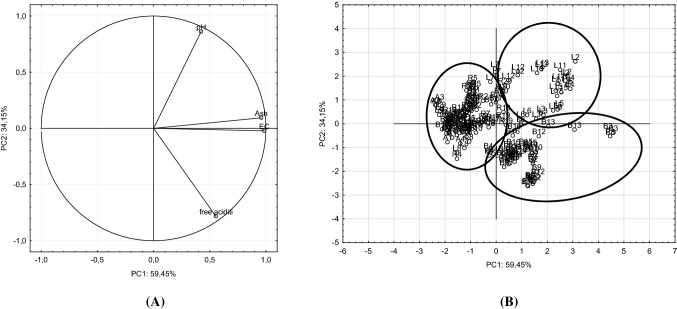
Representation of the variables (**A**) and honey samples (**B**) as functions of the PC1 versus PC2

Figure 3B presents the projection of the analyzed honeys onto a plane marked by two principal components PC1 and PC2, whereas Fig. 3A depicts projection of the studied variables onto the same plane. When analyzing the area marked by the two selected components it was observed that the investigated honeys could be divided into groups associated with their botanical origin (Fig. 3B). Three clusters of honeys were distinguished: linden, buckwheat and others, i.e. acacia and rapeseed. In the case of both linden and buckwheat honeys, single samples appeared outside the cluster characterizing a given group of honeys. Points depicting buckwheat honeys were in most cases highly concentrated in the cluster, which was indicative of a low internal variability of these samples. In turn, samples of linden honeys were more diversified, which was indicated by greater scattering of points in the area characteristic for this honey variety. Figure 3A shows that the most important parameters used in these analyses, which enabled botanical discrimination of honeys, was their electrical conductivity and ash content. The two other parameters (pH and content of free acids) played a less significant role in botanical identification of the analyzed honeys. Unfortunately, these parameters did not allow for botanical discrimination of the other honeys: acacia and rapeseed ones, because no separate clusters were distinguished for these varieties in the plane marked by PC1 and PC2. Points depicting these samples showed low internal variability, which was indicated by a high concentration within the cluster.

The analysis of correlation coefficients between variables and principal components (Table 2) and of projections of particular variables and the studied honeys onto the plane marked by the two first principal components (Fig. 3) enabled concluding that the key variables for the classification of the botanical origin of Polish nectar honeys could be electrical conductivity and ash content, followed by free acid content and pH of honeys. The importance of these parameters in the botanical classification of nectar honeys was also emphasized by authors from other countries (Silvano et al., 2014).

In conclusion, out of the 58 analyzed samples of honey, water content was exceeded in 13 samples—mainly of rapeseed honeys. Unfortunately, in the case of buckwheat honeys, the most unfavorable parameter turned out to be their free acidity as only two of the 17 analyzed honeys of this variety did not exceeded the permitted values set in legal regulations, which may be indicative of a low quality of honeys of this variety. A correlation was also analyzed between particular physicochemical parameters of honeys and a strong positive correlation (*r *= 0.934) was found between their ash content and electrical conductivity, i.e. parameters associated with the content of minerals. The strong positive correlation between electrical conductivity and ash content enables fast determination of total minerals content in honeys using conductometry. The chemometric analysis of the results of physicochemical parameter determinations demonstrated that electrical conductivity and ash content are also the most reliable markers in determining the botanical origin of linden and buckwheat honeys. Unfortunately, they appear insufficient for reliable identification of acacia and rapeseed honeys.
